# Cyclooxygenase-2 mediates microglial activation and secondary dopaminergic cell death in the mouse MPTP model of Parkinson's disease

**DOI:** 10.1186/1742-2094-3-6

**Published:** 2006-03-27

**Authors:** Rattanavijit Vijitruth, Mei Liu, Dong-Young Choi, Xuan V Nguyen, Randy L Hunter, Guoying Bing

**Affiliations:** 1 Department of Anatomy and Neurobiology, University of Kentucky, 800 Rose Street, Lexington, KY 40536, USA

## Abstract

**Background:**

Accumulating evidence suggests that inflammation plays an important role in the progression of Parkinson's disease (PD). Among many inflammatory factors found in the PD brain, cyclooxygenase (COX), specifically the inducible isoform, COX-2, is believed to be a critical enzyme in the inflammatory response. Induction of COX-2 is also found in an experimental model of PD produced by administration of 1-methy-4-phenyl-1,2,3,6-tetrahydropyridine (MPTP).

**Method:**

COX-2-deficient mice or C57BL/6 mice were treated with MPTP to investigate the effects of COX-2 deficiency or by using various doses of valdecoxib, a specific COX-2 inhibitor, which induces inhibition of COX-2 on dopaminergic neuronal toxicity and locomotor activity impairment. Immunohistochemistry, stereological cell counts, immunoblotting, an automated spontaneous locomotor activity recorder and rotarod behavioral testing apparatus were used to assess microglial activation, cell loss, and behavioral impariments.

**Results:**

MPTP reduced tyrosine hydroxylase (TH)-positive cell counts in the substantia nigra *pars compacta *(SNpc); total distance traveled, vertical activity, and coordination on a rotarod; and increased microglia activation. Valdecoxib alleviated the microglial activation, the loss of TH-positive cells and the decrease in open field and vertical activity. COX-2 deficiency attenuated MPTP-induced microglial activation, degeneration of TH-positive cells, and loss of coordination.

**Conclusion:**

These results indicate that reducing COX-2 activity can mitigate the secondary and progressive loss of dopaminergic neurons as well as the motor deficits induced by MPTP, possibly by suppression of microglial activation in the SNpc.

## Introduction

Parkinson's disease (PD) is a chronic and progressive motor disorder marked by degeneration of dopaminergic neurons in the substantia nigra *pars compacta *(SNpc). Increased inflammation and oxidative stress have been implicated in this neuronal death as elevated levels of cyclooxygenase-2 [1] and reactive microglia [2] have been found in PD brains. Cyclooxygenase, present as COX-1 and COX-2 isoforms, is the rate-limiting enzyme in arachidonic acid-derived prostaglandin production [3,4]. While COX-1 is constitutively expressed in most tissues, COX-2 is induced during pathophysiological responses to inflammatory stimuli such as bacterial endotoxin, interleukin-1 (IL-1), and various growth factors [5,6].

During the process of prostaglandin production, reactive oxygen species are generated as by-products [7] which, in addition to endotoxin, mitogens, cytokines, and certain inflammatory mediators, can activate microglia [8]. Microglia are also activated by oxidative stress [9]. Microglial activation causes the release of free radicals [10] and of inflammatory cytokines, including IL-1β, IL-6, and tumor necrosis factor-α [11]. Under normal circumstances, a response by microglia is protective in fighting off pathogens; however, under pathological conditions induced by certain insults – including oxidative stress, excitotoxicity from ion imbalance, and trauma – microglia can be over-stimulated and produce excessive cytotoxic agents that damage neurons, stimulating overexpression of neuronal and/or microglial COX-2 [1,10-17]. Co-propagation of COX-2 expression and microglial activation may cause secondary damage to neurons and the surrounding cellular environment; therefore, pharmacological intervention to stop the positive feed-back loop between COX-2 and microglial activation may prevent secondary injury induced by an excessive inflammatory response and oxidative stress.

In several epidemiological studies, nonsteroidal anti-inflammatory drugs have shown protective effects in reducing the risk of neurodegenerative disease such as Alzheimer's disease [8,18] and PD [19]. In the present study, we tested the hypothesis that excessive COX-2 aggravates MPTP-induced toxicity by perpetuation of the inflammatory response, which leads to secondary neuronal cell death in the SNpc. This study was designed to explore the role of COX-2-related inflammation in the pathogenesis of PD and to test the possibility of COX-2 inhibitors as a potential therapeutic drug for PD. Using an MPTP mouse model, C57BL/6N mice treated with therapeutic doses of valdecoxib showed improved cellular survival and behavioral function compared to vehicle controls. Similar results were obtained using COX-2-deficient mice. Both inhibition of COX-2 and genetic deficiency of COX-2 reduced SNpc microglial activation and mitigated MPTP-induced neurotoxicity on dopaminergic neurons in the SNpc.

## Materials and methods

### Animals and treatments

The development of COX-2 knockout mice has been previously described [20]. COX-2-deficient C57BL/6 mice were established at the National Institute of Environmental Health Science, Research Triangle Park, NC, USA, from which breeders were obtained to produce new breeding colonies at the University of Kentucky. Mice were kept on a 12:12 hour light:dark cycle and fed *ad libitum*. All COX-2 knockout (KO) -/-, heterozygous (HT) +/-, and wild type (WT) +/+ controls used were male littermates from a number of simultaneous matings and were seven to nine months old, weighing 25–35 grams. The genotype was determined by PCR. In addition to these sets of mice, male retired C57BL/6N breeders (aged seven to nine months, weighing 25–35 grams, Charles River Breeding Laboratories) were also used.

For each study, 8–12 mice per group received MPTP·HCL (Sigma-Aldrich, St. Louis, MO) at a dosage of 4 × 15 mg/kg i.p. at 1.5 hr intervals and were killed one or two weeks after the last injection. The non-MPTP treated controls received a comparable volume of 0.9% saline. MPTP handling and safety measures were in accordance with our Division of Laboratory Animal Resources Standard Operating Procedure and the Institutes of Health procedure for working with MPTP or MPTP-treated animals. Administration of valdecoxib (Bextra, Pharmacia, Chicago, IL) was modified from a published method [21]: 10, 30 or 50 mg/kg of valdecoxib was mixed with and administered as a cheese pellet, daily at 24-hour intervals from two weeks before MPTP injection until the end of the experiment. All procedures involving animals are approved by the Institutional Animal Care and Use Committee at the University of Kentucky and are in strict accordance with the National Institutes of Health *Guidelines for the Care and Use of Laboratory Animals*.

### Genotyping of COX-2-deficient mice

We performed genotyping with a standard protocol to identify wild-type, heterozygous, and homozygous *COX-2*-deficient mice. Four weeks after birth, segments of about three to five millimeters of mouse tails were digested with lysis buffer and proteinase-K at 55°C overnight (Genomic DNA purification kit, Gentra systems, Minneapolis, MN). After RNase treatment, DNA was separated by phenol-chloroform extraction and ethanol precipitation. PCR was performed with the following *COX-2 *specific primers (invitrogen, Carlsbad, CA):

COX-2 WT Forward: 5'-ACA CAC TCT ATC ACT GGC ACC-3'

COX-2 KO Forward: 5'-ACG CGT CAC CTT AAT ATG CG-3'

COX-2 Reverse: 5'-TCC CTT CAC TAA ATG CCC TC-3'

The thermal cycler (Eppendorf Mastercycler gradient, eppendorf, Hamburg, Germany) was programmed as follows: one cycle at 95°C for five minutes, and 30 cycles of 94°C for 30 seconds, 60°C for one minute, and 72°C for 90 seconds, followed by a final extension cycle of 72°C for seven minutes. PCR is expected to yield fragments of 760 and 900 bp for the *COX-2 *wild-type and knockout alleles, respectively.

### Immunohistochemistry

Brains were sectioned at 30 μm thickness on a sliding microtome for free-floating tissue sections. Every sixth section from a given area was stained with polyclonal antibodies (Ab) against neuronal TH (1:2000 Pelfreez, Roger, AR) or Mac-1 (1:1000 Serotec, Oxford, UK). Sections were incubated in 4% normal serum in PBS for 30 minutes. After this blocking step, the sections were incubated overnight in PBS containing 0.025% Triton X-100, 1% normal serum, and the primary antibodies at 4°Celcius. The avidin-biotin immunoperoxidase method with 3,3'-diaminobenzidine tetrahydrochloride as the chromagen was used to visualize immunoreactive cells (ABC Kits, Vector Laboratory, Burlingame, CA). For Nissl-staining, SNpc sections were stained with cresyl violet. Sections were then mounted on gelatinized slides, left to dry overnight, dehydrated in ascending alcohol concentrations, and mounted on Permount (Fisher Scientific, Fair Lawn, NJ).

### Western blot analysis

Cellular proteins were extracted from the striatal samples with an extract buffer containing 0.5% Triton X-100 and protease-inhibitor cocktail (1:1000, Sigma-Alsrich, St. Louis, MO). The tissues were homogenized in this buffer with the Fisher model 100 sonic dismembranator and put on ice for one hour. The soluble extracts were separated by centrifugation at 11,500 rpm for five minutes at 4°Celcius. Equal amounts of protein samples (20 μg) were mixed with the loading buffer (60 mM Tris-HCl, 2% SDS, and 2% β-mercaptoethanol, pH 7.2), boiled for 4 minutes, resolved by SDS-polyacrylamide gels, and transferred to a nitrocellulose filter (Millipore, Bedford, MA) using a semidry blotting apparatus (Bio-Rad Laboratories, Hercules, CA). After blocking with a solution containing 5% nonfat milk, the filters were incubated with TH (1:1000 Boehringer-Mannheim, Indianapolis, IN) or β-actin (Sigma, St. Louis, MO) antibodies for detection of the level of dopaminergic neuronal terminals, and for normalization of the loading protein. The signal was visualized by enhanced chemiluminescence according to the instructions of the manufacturer (Amersham Biosciences, Little Chalfont Buckinghamshire, England) by employing a goat anti-rabbit or goat anti-mouse secondary antibody conjugated with hydrogen peroxidase (Sigma-Aldrich, St. Louis, MO). Signal specificity was insured by omitting each primary antibody in a separate blot, and loading errors were corrected by measuring β-actin immunoreactive bands in the same membrane. The density measurement of each band was performed with Scion image software (Scion Corporation, Frederick, MD). Background samples from an equivalent area near each lane were subtracted from each band to calculate mean band density.

### Cell counting

The total number of TH- and Nissl-stained SNpc neurons and Mac-1-stained SNpc activated microglia were counted in sections from six to eight mice per group using the optical fractionator method for unbiased cell counting. The optical fractionator method of cell counting combines the optical dissector with fractional sampling, and is unaffected by the volume of reference (i.e., SNpc) or the size of the counted elements (i.e. neurons) [22]. Cell counts were performed by using a computer-assisted image analysis system consisting of a Zeiss Axioskop2Plus photomicroscope equipped with a MS-2000 (Applied Scientific Instrumentation, Eugene, OR) computer-controlled motorized stage, a Sony DXC-390 (Japan) video camera, a DELL GX260 workstation, and the Optical Fractionator Project module of the BIOQUANT Stereology Toolkit Plug-in for BIOQUANT Nova Prime software (BIOQUANT Image Analysis Corporation, Nashville, TN). Cell counting was done on both sides of SNpc of every sixth section throughout the entire extent of the SNpc. Each midbrain section was viewed at low power (× 10 objective), and the SNpc was outlined by using a set of anatomical landmarks. The cell numbers were counted at high power (× 40 objective). Adjacent sections immediately caudal and rostral to the sections used for TH staining were stained and counted for Nissl-stained neurons and Mac-1-stained activated microglia. TH- and Nissl-stained neurons were counted only when their nuclei were optimally visualized within one focal plane. Nissl-stained neurons were differentiated from non-neuronal cells by the clearly defined nucleus, cytoplasm, and a prominent nucleolus. After all of the cells were counted, the total numbers of neurons or activated microglia in the SNpc were automatically calculated by the module using the formula described by West et al. [22].

### Behavioral analysis and evaluation of locomotor activity

#### Apparatus

During the light period, locomotor activity was assessed using four automated activity chambers (Model RXYZCM-8, Accuscan Instruments, Columbus, OH). Each chamber consisted of a 41 × 41 × 31-cm^3 ^Plexiglas box with a grid of infrared beams mounted horizontally every 2.5 cm and vertically every 4.5 centimeters. The monitors were connected to a Digiscan Analyzer (Model DCM-8, Accuscan Instruments) that transmitted the number of beam breaks (activity data) to a computer. During operation, the pattern of beam interruptions was recorded for six consecutive 5-minute periods and analyzed by the computer.

#### Behavioral measures

Prior to valdecoxib administration, animals were allowed to habituate to the locomotor activity chambers during daily 30-min sessions over six consecutive days. Two measures of overall locomotor activity were obtained during the behavioral sessions: total distance traveled and vertical activity. Total distance traveled is quantified as the sum of the distance measured (in centimeters) across the 30-min recording period. Vertical activity is quantified as the sum of the number of vertical photobeam interruptions across the six 5-minute intervals.

### Rotarod testing

The Rotarod treadmill (MED Associates Inc, St. Albans, VT.), designed to measure motor performance and coordination, consists of a 3.6-cm diameter cylindrical treadmill connected to a computer-controlled stepper motor-driven drum that can be programmed to operate at a constant speed or in a defined acceleration mode. When the animal falls off the rotating drum, individual sensors at the bottom of each separate compartment automatically record the amount of time (in seconds) spent on the treadmill. Mice were trained two consecutive days before MPTP injections in acceleration mode (2–20 rpm) over five minutes. The training was repeated with a fixed speed (16 rpm) until the mice were able to stay on the rod for at least 150 seconds. On day 2, 4, and 6 after MPTP injections, mice were assessed for their coordination capability with a maximum recording time of 150 seconds. Rotational speeds of 16, 20, 24, 28, and 32 rpm were recorded in succession, and the overall rod performance (ORP) for each mouse was calculated by the trapezoidal method as the area under the curve in the plot of time on the rod versus rotation speed [23].

### Statistical analysis

All data were analyzed using an IBM-based personal computer statistical package (SYSTAT 10, SPSS Inc, Chicago, IL). Except for the correlation analyses, all values are expressed as the mean ± SEM, and differences among means were analyzed by using one- or two-way analysis of variance (ANOVA) with time, treatment, or genotype as the independent factor. When ANOVA showed significant differences, pairwise comparisons between means were tested by Bonferroni *post hoc *testing. Statistical significance was set at p < 0.05 for all analyses.

## Results

### Valdecoxib treatment attenuates MPTP-induced dopaminergic neurodegeneration

**Figure 1:**
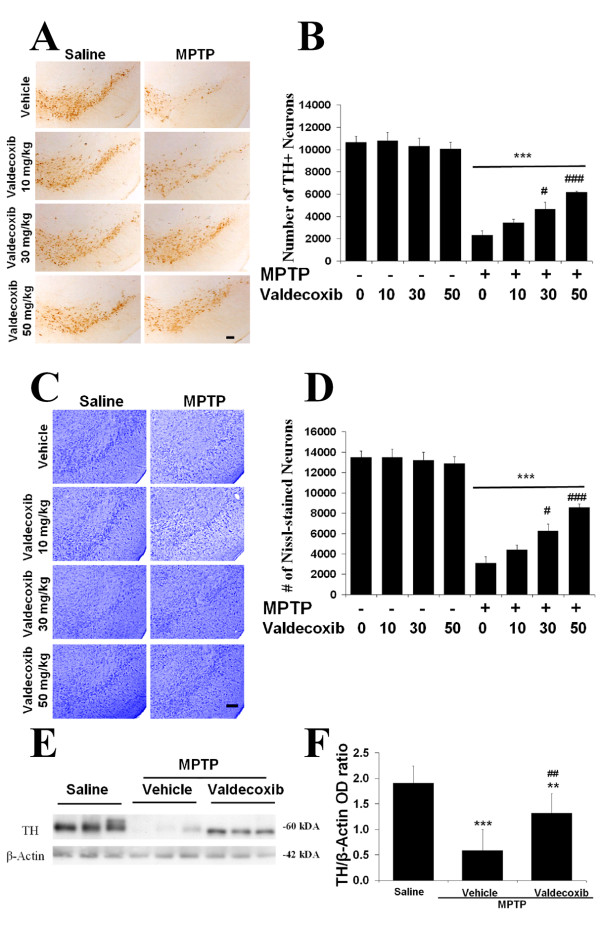
**TH-positive neurons are more resistant to MPTP in mice treated with a selective COX-2 inhibitor (valdecoxib). A: **Photomicrographs of representative SNpc sections stained with an antibody against TH. The SNpc tissues were collected 14 days post-MPTP injection. The MPTP (4 × 15 mg/kg, 1.5 hr interval, i.p.)-treated mice have fewer TH-positive neurons compared to the saline groups. However, valdecoxib treatment reduced the neuronal loss, especially at a higher dose (30 or 50 mg/kg daily). **B: **MPTP administration led to significant loss of TH-positive neurons numbers by 78 percent for vehicle and by only about 68, 56, and 42 percent for 10, 30 and 50 mg/kg valdecoxib-treated mice, respectively. Among the MPTP-injected mice, the valdecoxib-treated mice had 10 to 32 percent more TH-positive neurons than the vehicle-treated mice. Nissl stain shows similar trends (**C&D**). **E: **Inhibition of COX-2 reduced the MPTP-induced loss of the striatal TH immunoreactivity. **F: **After MPTP treatment, the vehicle-treated mice had significantly reduced TH immunoreactivity compared to the saline-treated mice (***p < 0.001). Among the MPTP-injected mice, the valdecoxib (30 mg/kg daily)-treated mice had at least 30% higher TH immunoreactivity than the vehicle-treated mice. Data are means ± SEM for six to eight mice per group, **p < 0.01 and ***p < 0.001 compared to saline+vehicle group; #p < 0.05, ##p < 0.01 and ###p < 0.001 compared to MPTP+vehicle group, by ANOVA with subsequent Bonferroni for multiple comparisons. Scale bar, 100 μm.

To determine the neuroprotective effect of COX-2 inhibitors against MPTP-induced neurotoxicity, TH-positive and Nissl-stained neurons in the SNpc were stereologically quantified. Treatment with valdecoxib did not affect the number of TH-positive and Nissl-stained neurons in the SNpc (Fig. 1A &1C), and a stereological analysis showed no significant difference among the saline-injected groups (Fig. 1B &1D). Fourteen days after the MPTP injections, there was a clear MPTP-associated toxic effect on the SNpc as revealed by diminished TH- and Nissl-stained neurons in sections from MPTP-treated mice, and the loss was significantly reduced by treatment with valdecoxib (Fig. 1A). Treatment with MPTP induced about 78% TH-positive cell loss while the various valdecoxib pretreatment groups showed only about 42–68% loss of TH-positive neurons. Compared to the saline+vehicle control, there was significant loss of TH-positive neurons in the MPTP-treated groups (***p < 0.001) (Fig. 1B). The numbers of TH-positive neurons remaining in the SNpc after MPTP injection in the higher doses of valdecoxib-treated mice (30 and 50 mg/kg) are statistically higher than in the MPTP+vehicle group (#p < 0.05 and ###p < 0.001, Fig. 1B). Nissl stain showed similar trends and statistical results (Fig. 1C &1D), suggesting an actual TH-positive neuronal loss instead of a loss of TH expression. To determine whether valdecoxib could prevent not only MPTP-induced loss of SNpc dopaminergic neurons but also the loss of striatal dopaminergic fibers, we assessed the density of TH immunoreactivity in the striata of the different mice (Fig. 1E). MPTP significantly reduced striatal TH immunoreactivity compared to the saline control by 70% in the MPTP+vehicle (***p < 0.01) and only about 30% in the MPTP+valdecoxib mice (Fig. 1F). These findings demonstrate that valdecoxib protects the nigrostriatal pathway against the MPTP-induced degeneration of dopaminergic neurons.

### COX-2 deficiency attenuates MPTP-induced loss of TH-positive neurons and neuronal terminals in the nigrostriatal system

**Figure 2:**
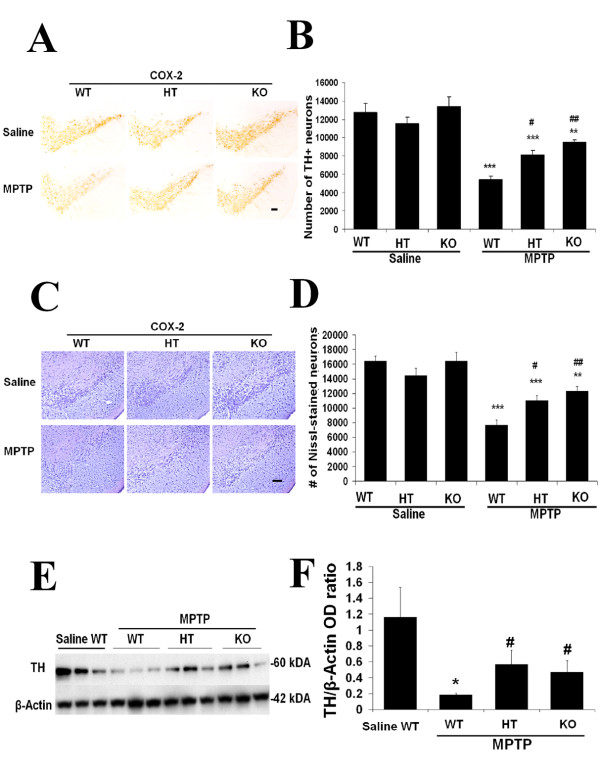
**Ablation of COX-2 protects TH+ Neurons in SNpc from MPTP. A: **In COX-2-/- (KO) mice, dopaminergic neurons are protected from MPTP neurotoxicity. Representative TH immunocytochemistry shows a marked loss of TH-positive neurons in SNpc of COX-2 +/+ (WT) mice compared to both COX-2 -/- (KO) and COX-2 +/- (HT) mice after MPTP treatment. **B: **MPTP treatment leads to a significant loss in number of TH+ neurons. TH-positive cells in the SNpc were bilaterally counted under 40 × objective. Nissl stain shows similar trends (**C&D**). **E: **COX-2 deficiency reduced the MPTP-induced loss of striatal TH immunoreactivity. **F: **MPTP-treated WT mice had significantly reduced TH immunoreactivity compared to the saline WT (*p < 0.05). Among the MPTP-treated mice, the COX-2-deficient mice had at least 30% higher TH immunoreactivity than the WT mice. Data are means ± SEM for six to eight mice per group, *p < 0.05, **p < 0.01 and ***p < 0.001 compared to saline+vehicle group; #p < 0.05 and ##p < 0.01 compared to MPTP+vehicle group, by ANOVA with subsequent Bonferroni for multiple comparisons. Scale bar, 100 μm.

Because of possible unintended confounding effects associated with oral administration of COX-2 inhibitors [24], we validated the pharmacological approaches using a genetic approach with COX-2-deficient mice. Immunohistochemical studies revealed no differences between saline-treated mice of different COX-2 genotypes (Fig. 2A). However, among the MPTP-treated mice, the COX-2 knockout (KO) mice exhibited the least TH-positive cell loss, while the wild-type (WT) mice had the most loss. The heterozygous (HT) mice showed TH-positive neuronal survival comparable to the KO mice. MPTP significantly reduced the number of the TH-positive neurons in the SNpc, and both the HT and KO mice had significantly more (20–30%) TH-positive neurons than the WT mice (#p < 0.05 and ##p < 0.01, respectively). Nissl staining showed similar trends and statistical results (Fig. 2C &2D), which indicates a true loss of the TH-positive neurons rather than a decrease in TH expression. To determine whether deleting the *cox*-2 genes can prevent MPTP-induced SNpc dopaminergic neuron loss as well as the loss of striatal dopaminergic fibers, we assessed the TH immunoreactivity in striata from the different groups of mice by Western blot analysis (Fig. 2E). MPTP significantly reduced striatal TH immunoreactivity compared with the control by 80% in the WT (*p < 0.05) but by less than 50% in the HT and KO mice. Compared to the MPTP-treated WT mice, both the MPTP-teated HT and KO mice had statistically higher striatal TH immunoreactivity (#p < 0.05, Fig. 2F). These results support the effects of dopaminergic neuronal protection observed with the selective COX-2 inhibitor valdecoxib and demonstrate consistency between the pharmacological and genetic approaches.

### The selective COX-2 inhibitor valdecoxib or COX-2 deficiency abates microglial activation

**Figure 3:**
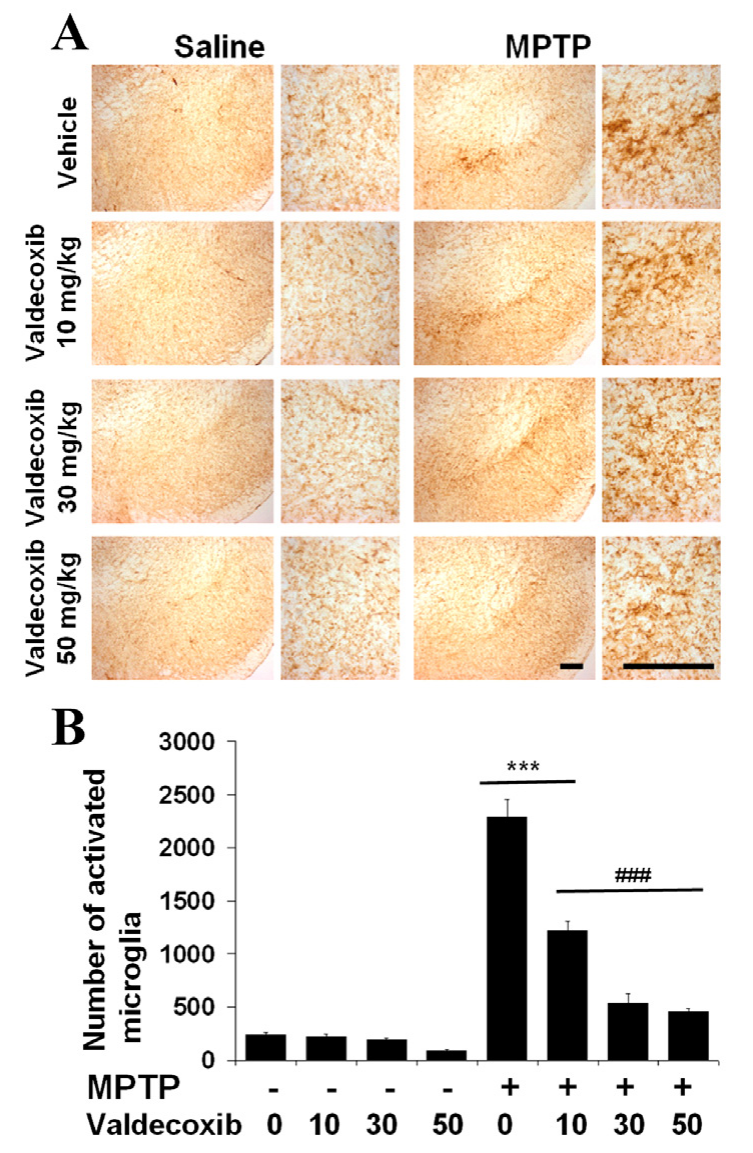
**MPTP-induced microglial activation is inhibited by the selective COX-2 inhibitor valdecoxib. A: **At 14 days post-MPTP injection, there was a high level of microglial activation in the SNpc. Unlike the vehicle group, mice treated with valdecoxib have diminished microglial activation. Pictures on the right are magnified photographs of the pictures on the left side. In contrast to inactivated striped microglia in MPTP+valdecoxib and control saline sections, activated microglia in MPTP+vehicle sections have a rounder body, fatter processes and denser stain. **B: **MPTP treatment leads to a significant increase in the number of activated microglia in mice receiving vehicle or the lowest dose of valdecoxib (10 mg/kg daily) but not the higher dose of valdecoxib (30 or 50 mg/kg daily). Activated microglia in the SNpc were bilaterally counted under a 40 × objective. Data are means ± SEM for six to eight mice per group, ***p < 0.001 compared to saline+vehicle group; ###p < 0.001 compared to MPTP+vehicle group, by ANOVA with subsequent Bonferroni for multiple comparisons. Scale bar, 100 μm.

To investigate potential mechanism of secondary dopaminergic neuronal death, we performed immunohistochemistry using a microglia-specific antibody (anti-Mac-1 antibody). A large number of activated microglia, which had expanded cell bodies and poorly ramified short and thick processes, were seen in the MPTP+vehicle group but were not observed in the MPTP+valdecoxib group or saline-treated groups (Fig. 3). This supports our hypothesis that inhibition of COX-2 expression during injury stimuli (MPTP) can reduce microglial activation, which may lead to secondary degeneration and progressive cell loss. In sections with numerous activated microglia, the SNpc can be distinguished from the surrounding areas as the activated microglia tend to stay within the SNpc or along the border of the SNpc. MPTP-induced microglial activation was clearly observed in the vehicle-treated mice, but to a lesser extent in the 10, 30 or 50 mg/kg valdecoxib-treated mice (Fig. 3A). Some activated microglia could be seen in the saline-treated animals, but the number of activated microglia was very small compared to the MPTP-treated WT mice (***p < 0.001, Fig. 3B).

**Figure 4:**
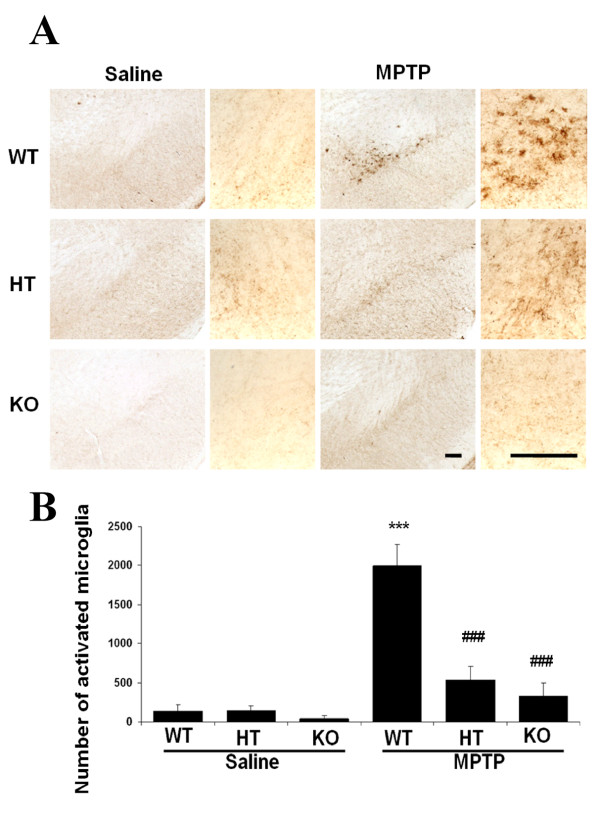
**Ablation of COX-2 reduces MPTP-induced microglial activation 7 days post-MPTP injection. A: **Seven days after MPTP treatment, COX-2 +/+ mice had a local increase of microglial activation in the SNpc, which is shown with immunohistochemical stains for Mac-1, compared to saline-treated or MPTP-treated COX-2 +/- and -/- mice. The magnified right panels show activated microglia. In contrast to inactivated striped microglia in MPTP-treated COX-2-deficient and control saline sections, activated microglia in MPTP-treated COX-2 wild-type have a rounder body, fatter processes and denser stain. **B: **MPTP treatment leads to a significant increase in the number of activated microglia in the WT relative to the HT and KO mice. Activated microglia in the SNpc were bilaterally counted under a 40 × objective. Data are means ± SEM for six to eight mice per group, ***p < 0.001 compared to saline+vehicle group; ###p < 0.001 compared to MPTP+vehicle group, by ANOVA with subsequent Bonferroni for multiple comparisons. Scale bar, 100 μm.

To further evaluate the role of COX-2 in modulating microglial activation in a COX-2 dose-responsive manner, we performed Mac-1 staining and counted the number of activated microglia in COX-2-deficient mice receiving only saline. Saline-treated mice showed no differences among the COX-2 WT, HT and KO genotypes. MPTP-induced microglia activation was again observed in the WT mice but was comparatively less in the HT or KO mice (Fig. 4A). The numbers of activated microglia in the MPTP-injected HT and KO mice were significantly (four and five times) lower than the MPTP-injected WT mice (###p < 0.001, Fig. 4B). These findings suggest that COX-2 may play a role in modulating microglial activation.

### Dopaminergic neuronal survival is inversely correlated to COX-2 and microglial activation

**Figure 5:**
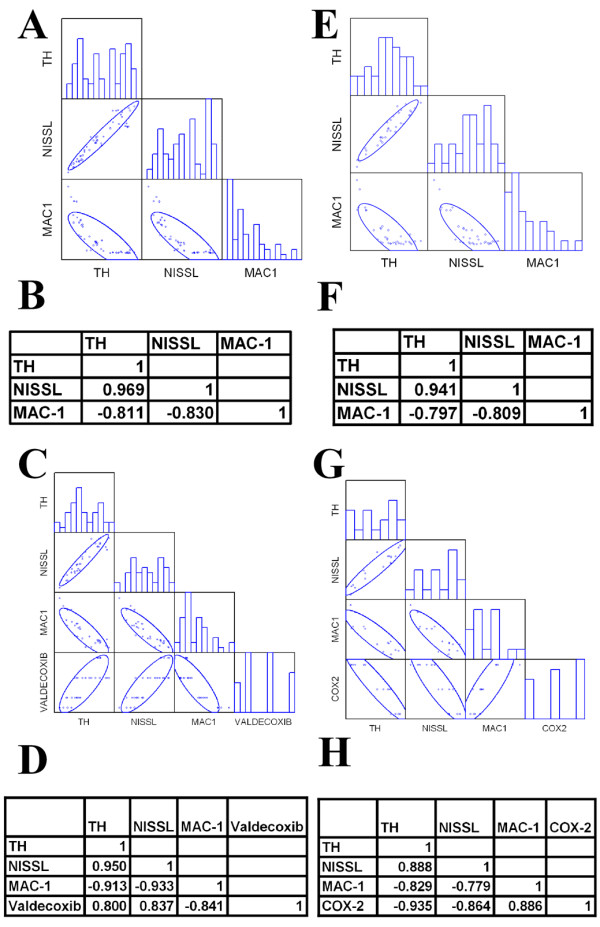
**TH-positive neuronal counts have strong negative correlation with the number of Mac-1-stained activated microglia and COX-2. **Figures 5A-D show results from the study from valdecoxib-treated mice and Figures 5E-H show results from the study from COX-2-deficient mice. The results from the correlation matrix shown in panels **A **and **E **are tabulated in panels **B **and **F**, respectively. As expected, the number of viable TH-positive neurons was strongly correlated with the number of neurons counted with the Nissl stain (r > 0.90). Importantly, the number of activated microglia was strongly negatively correlated with TH- and Nissl-stained neurons, both r ≈ -0.80 (p < 0.05, Pearson correlation test, n = 6–8 per group). The result from the correlation matrix shown in **C **is indicated in **D**. Similar analysis as in **A **and **B **was used, but included only MPTP-treated values and assigned value of 0 for no treatment and 10, 30 and 50 for increasing dosage of valdecoxib. The results were similar to those of **A **and **B **with a positive correlation of the amount of daily valdecoxib to TH- and Nissl-stained neuronal numbers (both r_s _≥ 0.80) and a strong negative correlation of the dosage of valdecoxib to microglial activation (r_s _= -0.841, p < 0.05 Spearman correlation statistic, n = 6–8 per group). The result from the correlation matrix shown in **G **is indicated in **H**. A similar analysis as in **E **and **F **was used, but included only MPTP-treated values and assigned values of 1.0, 0.5 and 0.0 to COX-2 WT, HT and KO, respectively. The results were similar to those of **E **and **F **with strong negative correlation of COX-2 to TH- and Nissl-stained neuronal numbers (both r_s _≈ -0.90) and strong positive correlation of COX-2 to microglial activation (r_s _= 0.886, p < 0.05 Spearman correlation statistic, n = 6–8 per group).

To determine the relationship between the TH-positive neurons and microglia activation, we performed immunohistochemistry of adjacent SNpc sections of each brain, counted the cell numbers and studied statistical correlations among them. The sections counted for dopaminergic neurons (Fig. 1A &1B or Fig. 2A &2B) were 30 μm caudal relative to the sections evaluated for Mac-1 immunoreactivity (Fig. 3 or Fig. 4) and 30 μm rostral relative to the Nissl-stained sections (Fig. 1C &1D or Fig. 2C &2D) of the same mouse brain. The Pearson correlation matrix shows the graphic representation of pooled data values for the number of TH- and Nissl-stained neurons and Mac-1-stained activated microglia of each mouse from the valdecoxib study (Fig. 5A) and from the COX-2 deficiency study (Fig. 5E). TH-positive neuron counts were highly correlated with Nissl-stained neuron counts (r > 0.94), while microglial activation had strong negative correlations with both TH- and Nissl-cell counts (both with Pearson correlation statistic r ≈ -0.80; p < 0.05; Fig. 5B and Fig. 5F). These results suggest a strong co-existence of progressive dopaminergic neuronal degeneration with activation of microglia.

The relationship of COX-2 inhibition or expression to the TH-positive neuronal survival and microglia activation can be inferred from Figures 1 and 3 as well as Figures 2 and 4. Statistically, the correlation of COX-2 to the number of TH- and Nissl-stained neurons and Mac-1-stained activated microglia can be determined by the correlation analysis. We ranked the data as 0, 10, 30 or 50 in accordance with the mg/kg amount of valdecoxib each mouse received daily. It has been suggested that the level of COX-2 in the HT mouse is half of the WT [25]; therefore, we assigned the WT as having a full expression capability of COX-2 and designated the amount of COX-2 in the WT, HT and KO mice as 1.0, 0.5 and 0.0, respectively. In Fig. 5C &5D and Fig. 5G &5H, the correlation was analyzed as in Fig. 5A &5B and Fig. 5E &5F only using the MPTP-treated samples where COX-2 and microglial activation is induced by MPTP [1]. From the COX-2 inhibitor study (Fig. 5C &5D), the number of TH-positive neurons was strongly positively correlated with the number of Nissl-stained neurons and the amount of the COX-2 inhibitor administered per day (r_s _≈ 1.0 and r_s _= 8.0, respectively). There was also a strong negative correlation with the Mac-1-stained reactive microglia (r_s _≈ -0.90, p < 0.05, Spearman correlation statistic, Fig. 5D). It is important to note that the Mac-1-stained, reactive microglia counts, showed a strong negative correlation with the level of the COX-2 inhibitor (r_s _≈ -0.80, p < 0.05, Spearman correlation statistic, Fig. 5D). These results imply that decreased activity of COX-2, due to inhibition by valdecoxib, strongly correlates with higher survival of dopaminergic neurons and decreased microglial activation. Similar results were obtained in the COX-2 deficiency study (Fig. 5G &5H), which showed that the number of TH-positive neurons strongly positively correlated with the number of Nissl-stained neurons (r_s _≈ 0.9) and strongly negatively correlated with the number of Mac-1-stained reactive microglia and with the level of COX-2 (r_s _≈ -0.80 and r_s _≈ -0.90, respectively, p < 0.05, Spearman correlation statistic, Fig. 5H). Mac-1-stained reactive microglia counts strongly positively correlate with the level of COX-2 (r_s _≈ 0.90, p < 0.05, Spearman correlation statistic, Fig. 5H). These results indicate a strong correlation of progressive dopaminergic neurodegeneration with increased COX-2 and increased microglial activation.

### Valdecoxib reduces MPTP-induced locomotor activity deficits

**Figure 6:**
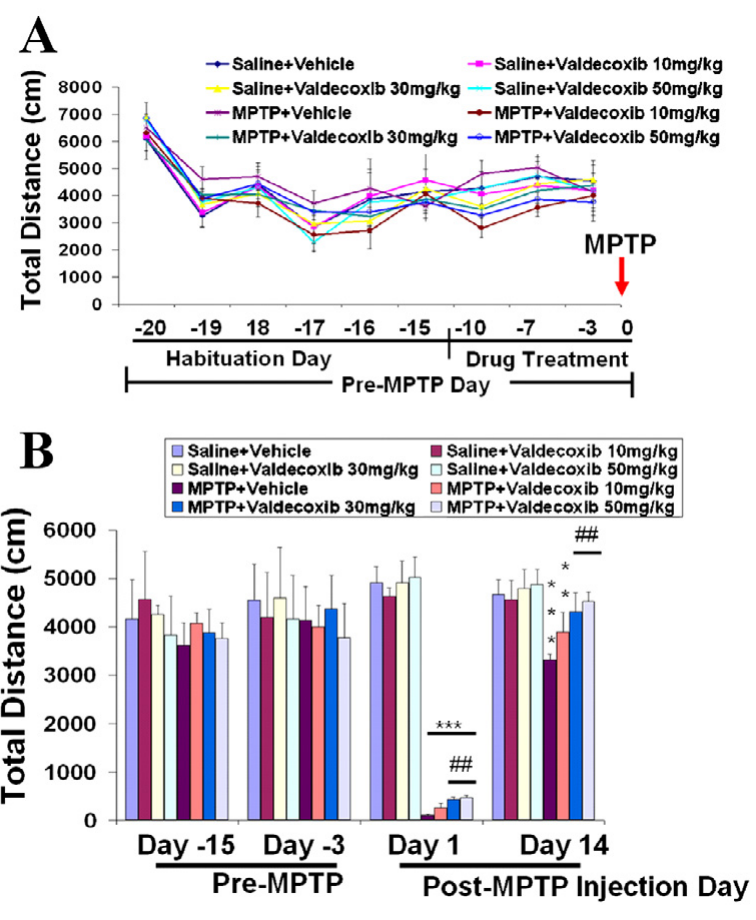
**Selective COX-2 inhibitors alleviate MPTP-induced loss of spontaneous locomotor activity of C57BL/6 mice. **On pre-MPTP days (**A**), there were no statistically significant differences among the experimental groups in the total distance (cm) mice traveled during each 30-min session. By the end of the pre-MPTP treatment period (3 days before MPTP injection), mice in all groups traveled similar distances. MPTP was administered at day zero. After MPTP treatment (**B**), mice initiated less spontaneous locomotor activity than they had prior to MPTP administration. On average, valdecoxib-treated mice performed up to 25% better than the vehicle-treated mice. Data are means ± SEM for eight to twelve mice per group pre-MPTP treatment and six to eight mice per group post-MPTP treatment. Statistical significance was assessed by two-way repeated measures ANOVA with Bonferroni *post hoc *test, **p < 0.01 and ***p < 0.001 compared to the saline+vehicle group; ##p < 0.01 compared to MPTP+vehicle group.

**Figure 7:**
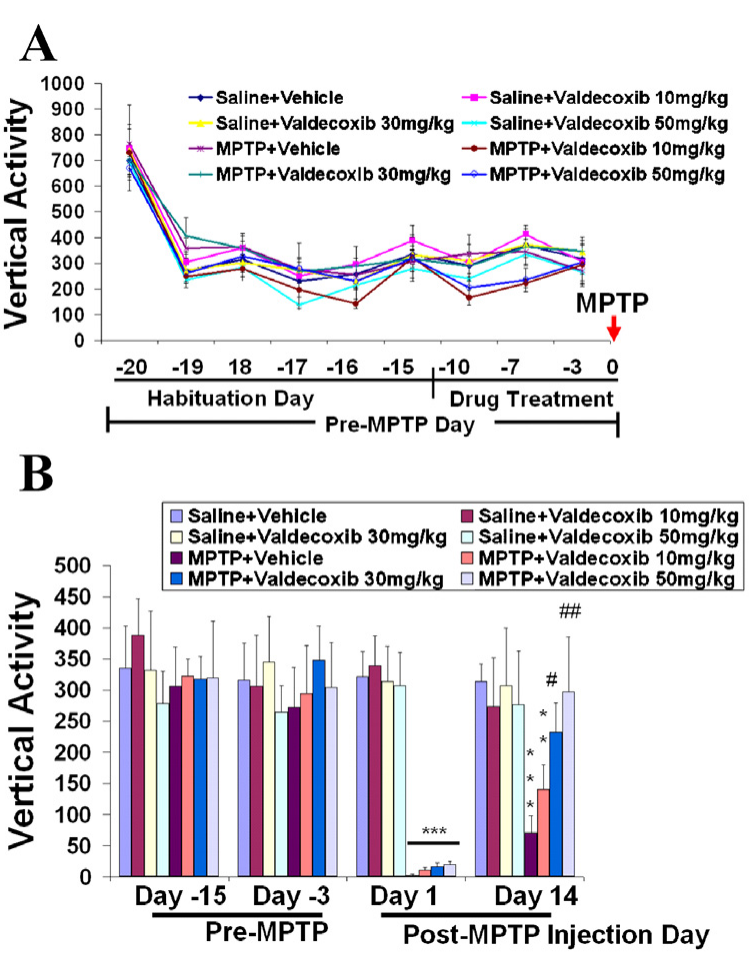
**MPTP-induced deficit in vertical activity is decreased by selective COX-2 inhibitors. **An objective measure of the vertical activity, recorded by an automated locomotor activity testing machine, revealed the ability of valdecoxib to maintain rearing activity closer to that of control-saline-treated mice (B) and to their baseline prior to MPTP injection (A). In agreement with the total spontaneous horizontal distance results, vertical activity of valdecoxib-treated mice was statistically less impaired. Data are means ± SEM for eight to twelve mice per group pre-MPTP treatment and six to eight mice per group post-MPTP treatment, and were analyzed by two-way repeated measures ANOVA with Bonferroni *post hoc *test, *p < 0.05 and ***p < 0.001 compared to the saline+vehicle group; #p < 0.05 and ##p < 0.01 compared to MPTP+vehicle group.

To determine the behavioral manifestations of the observed cellular changes, we assessed spontaneous locomotor activity by measuring the total distance traveled and vertical activity for 30 minutes using an automated open-field activity apparatus. There were no differences among the mice before MPTP injection (before day 0, Fig. 6A &7A), and horizontal distance and rearing frequency were similar among all groups. A sharp reduction in distance from the first day of the behavioral testing (day -20) to a lesser but consistent baseline level at later days (from day -19), indicated a habituation effect (Fig. 6A &7A). Two weeks (day -14) before MPTP injection, Valdecoxib treatment was started. To determine the effect of valdecoxib on pre-MPTP mouse behavior, mice were tested three times during valdecoxib treatment alone (day -10, -7 and -3) and showed no differences from non-valdecoxib-treated groups or their own baselines (the distance traveled during day -17 to day -15). On day zero, mice were injected with MPTP or saline. At day 1 post-MPTP injection (Fig. 6B &7B), a marked reduction in spontaneous activity was observed (***p < 0.001). However, the reduction of spontaneous activity was likely due to systemic MPTP side-effects. Nevertheless, on day 1, the valdecoxib-treated mice, especially the ones treated with higher dosages of 30 or 50 mg/kg per day, were able to initiate spontaneous locomotor activity more than the vehicle-treated mice (##p < 0.01, Fig. 6B). Behavioral performance was tested on multiple days to ensure the validity of the results. Our previous studies revealed that MPTP-treated mice recovered from MPTP-induced hypoactivity after a few days, and their locomotor activity fluctuated and then stabilized around two weeks (unpublished observation). At two weeks post-MPTP injection, 30 or 50 mg/kg valdecoxib-treated mice recovered to the level of saline controls while the MPTP-treated vehicle group remained hypoactive (***p < 0.001) and had a statistically lower total ambulating distance and decreased rearing frequency compared to the valdecoxib-treated group (##p < 0.01, Fig. 6B and #p < 0.05 and ##p < 0.001, Fig. 7B, respectively). These behavioral results reflect the cellular protective effects of the COX-2 inhibitor when provided in sufficient amounts.

### MPTP-induced loss of locomotor coordination is alleviated in COX-2-deficient mice

**Figure 8:**
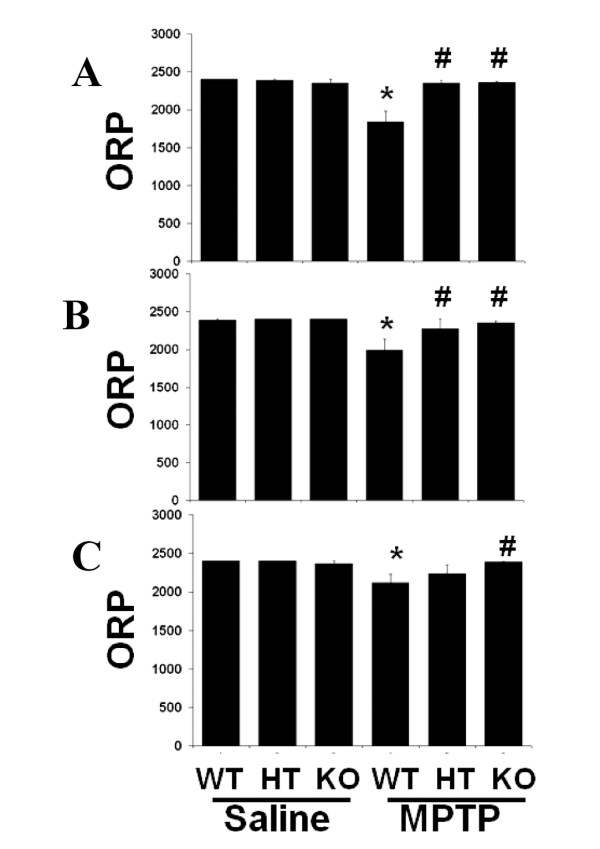
**COX-2 deficiency showed a protective effect against motor deficit in MPTP-injected mice. **Animals were trained on the rod for two consecutive days before intraperitoneal MPTP (4 × 15 mg/kg, 1.5 hr interval) or saline injection. Mice were assessed for their Rotarod performance on day 2 (**A**), 4 (**B**) and 6 (**C**) after MPTP injection. Motor deficit is observed in the MPTP-treated animals, but deficiency in COX-2 significantly prevents this impairment. Deficiency in COX-2 does not affect baseline motor function in the saline-injected COX-2 HT and KO mice. Data are means ± SEM for six to eight mice per group post-MPTP treatment and analyzed by two-way repeated measures ANOVA with Bonferroni *post hoc *test, *p < 0.05 compared to the saline+vehicle group and #p < 0.05 compared to MPTP+vehicle group.

To characterize behavior resulting from underlying biological changes in the COX-2-deficient mice, we assessed locomotor coordination and balance in 150-second sessions using an automated Rotarod testing apparatus. All mice were trained and were able to stay on the rotating rod at a fixed speed of 16 rpm for 150 seconds. Two days after MPTP injection (Fig. 8A), the WT mice spent significantly less time overall on the rotating rod at various speeds. This is reflected in a low overall rod performance (ORP) score (*p < 0.05), which implies a loss of motor coordination. The COX-2-deficient mice significantly retain postural balance at several speeds for longer periods of time than the WT mice (#p < 0.05). For both the MPTP-treated HT and KO mice, the ORPs appeared to be similar to the saline controls. After day 4, statistical significance for the difference in ORP between HT and WT mice was lost. On day 4 (Fig. 8B) and day 6 (Fig. 8C), the HT mouse performance was between that of the WT and the KO mice. The sensitivity of this assay may be suboptimal due to a ceiling effect from the maximum trial length of 150 seconds, the ability of some coordination-impaired mice to cling onto the rotating treadmill without falling, and nonspecific effects of high rotation speeds on both impaired and control mice. The saline WT mice and the MPTP KO mice remain relatively stable with respect to time spent on the rod at the different speeds throughout the experiment. These behavioral data implicate a beneficial effect of reduced COX-2 levels in COX-2-deficient mice.

## Discussion

The major finding of this study is that the selective COX-2 inhibitor valdecoxib or deficiency of COX-2 inhibits microglial activation and protects the nigrostriatal dopaminergic system against MPTP-induced neurotoxicity and behavioral deficits. This study was conducted in attempt to fill in the gap within the literature on the effects of COX-2 inhibition in protecting SNpc dopaminergic neurons from MPTP-induced neurotoxicity. We inhibited COX-2 using both pharmacological and genetic approaches in aged mice (7–9 months old). The HT mice were included to determine the effects of heterozygosity for COX-2 on MPTP-induced cellular toxicity and behavioral impairments. This is because COX-2 is an inducible enzyme, and MPTP may or may not be able to induce the same magnitude of COX-2 in WT mice as in heterozygous mice. We are the first to show a potential link between COX-2 and microglial activation in COX-2 heterozygous mice in the demonstration of correlations between the numbers of TH- and Nissl-stained neurons, Mac-1-stained microglia, and of all genotypes or the three inhibitor dosages of COX-2. We also show that the behavioral benefits of COX-2 inhibition and deficiency correlate well with the observed cellular protection.

Non-biased stereological cell counting indicated that MPTP-treated mice have severe loss of TH-positive neurons in the SNpc while valdecoxib-treated mice, in a dose-responsive manner, have reduced MPTP-associated degeneration of dopaminergic neurons. The concordance between the reduction of TH expression and neurodegeneration was made distinctive by the neuronal loss revealed by quantification of Nissl-stained neurons from adjacent sections caudal to the TH-stained sections. Thus, it appears that selective COX-2 inhibition can attenuate MPTP-induced dopaminergic neurodegeneration. This result is supported by other investigators that used slightly different experimental setups [1,26,27]. To ensure that the results obtained with the COX-2 inhibitor are specifically related to COX-2, we conducted an analogous experiment with COX-2-deficient mice. In concurrence with other laboratories [1,28], COX-2 deficiency protects TH-positive neurons in the SNpc from MPTP-induced neurotoxicity. The efficacy of an inhibitor to inhibit COX-2 is likely to give a result intermediate to that of the COX-2 heterozygous (HT) or knock-out (KO) mice. The TH-stained neuronal bodies in the SNpc were protected as well as the striatal TH-stained fibers/terminal, which suggests protection of the nigrostriatal pathway.

Mounting evidence has demonstrated induction of microglial activation in neurodegenerative diseases [11,16,29], including PD [9,30] and PD animal models [26,31]. It is controversial whether such activation of microglia is beneficial or detrimental to neurons. Our research sheds light on the role of activated microglia in neurodegeneration, as we observed the neuroprotection afforded by selective COX-2 inhibition or deficiency of COX-2, which correlated with attenuation of microglial activation. Using selective COX-2 inhibitors, earlier investigators reported either no inhibition of activated microglia in the mouse MPTP-induced PD model [1] or decreased activation of microglia in the rat 6-hydroxy dopamine-induced PD model [32]. We show for the first time a direct correlation between COX-2 and microglia activation in the mouse MPTP model. Using the optical fractionator method to estimate total cell number, we found a substantial decrease in the activation of microglia within the SNpc in MPTP-treated COX-2 HT mice and an even further reduction in KO mice relative to the MPTP-treated WT mice. From these results we speculate that COX-2 may play some role in suppressing the chronic inflammation and microglial activation that is observed years after MPTP exposure [33-35]. This secondary inhibition of microglia activation will be expected to attenuate the progressive cell loss induced by the inflammatory response.

Using adjacent SNpc sections to stain and count the number of TH- and Nissl-stained neurons and Mac-1-stained activated microglia, we performed a correlation analysis of TH- and Nissl-stained neurons, COX-2 and Mac-1-stained activated microglia. With all the data analyzed or with only the data from the MPTP-treated animals, we can see that the number of TH-stained neurons has a high positive correlation with the number of Nissl-stained neurons. This implies that the lower TH count is due to degeneration of neurons rather than to reduction of TH expression. The strong negative correlation of Mac-1 to TH- or Nissl-stained neurons, with or without data from the non-MPTP-treated animals, implicates a high number of activated microglia from an MPTP insult co-exists with dopaminergic neuronal cell death. From the valdecoxib study, the number of TH-positive neurons was strongly correlated with the number of Nissl-stained neurons and the magnitude of COX-2 inhibition, but had a strong negative correlation with the Mac-1-stained reactive microglia. Thus, inhibition of COX-2 correlates with reduced degeneration of the dopaminergic neurons. We demonstrate a clear correlation of COX-2 gene expression to the number of Mac-1-stained, activated microglia or to the degeneration of dopaminergic neurons (inferred from TH and Nissl). The WT, HT and KO animals with full (1.0), half (0.5) and zero (0.0) availability of COX-2 had a linear positive relationship with the number of activated microglia (Mac-1) but had an inverse relationship with the numbers of TH- and Nissl-stained neurons. In addition, Mac-1-stained reactive microglia counts strongly positively correlate with COX-2 levels. These results suggest that inhibition or deficiency of COX-2 correlates well with the amount of microglial activation and with the degeneration of the SNpc dopaminergic neurons that result from MPTP-induced neurotoxicity.

Animal behavior is a result of underlying cellular physiology, and the results observed at the cellular level are augmented by the behavioral analysis. We thoroughly assessed motor movement using an automated locomotor activity test and Rotarod apparatus. For the total distance and vertical activity assessment, mice were able to habituate to the testing protocol, and the results from the habituation period suggest that mice adjust to the setup very quickly and that the habituated level of activity is much lower than the first-time exposure. Once adjusted, the level of spontaneous horizontal travel and vertical activity were constant for non-MPTP treated animals throughout the experiment and for all animals prior to MPTP injection. Moreover, valdecoxib does not affect the movement of mice pre-MPTP treatment. Our results differ from those of other investigators who have only reported behavioral testing one day post-MPTP injection or earlier [27,36-39]. This is likely because we included behavioral measurements at two weeks when the locomotor activity of each mouse became stable and was more consistent within each experimental group. Our present study demonstrates that MPTP significantly reduces total distance traveled and vertical activity of the mice, and that valdecoxib decreases MPTP-induced behavioral impairments.

MPTP-induced loss of locomotor balance and coordination was measured using the Rotarod test after the mice were able to habituate to the machine and the procedure. Pre-training ensured that all the mice could walk on the rod for at least 150 seconds at 16 rpm. As a result, the performance differences we observed post-MPTP injections were unlikely due to individual differences in adapting to a new environment or in strength, but were rather due to the MPTP-induced loss of locomotor coordination or balance. Consistent with the protection seen at the cellular level, the COX-2 HT mouse performance was intermediate to the WT and KO, although the HT mice behaved generally closer to the KO than the WT mice. The heterozygous mice have half the WT level of COX-2 [25], and this may explain the pharmacological benefits of reducing COX-2 activity because they exhibit less MPTP-induced Parkinson's disease-like pathology and symptoms as shown in this study. Therefore, it is reasonable to infer that 50% inhibition of COX-2 activity may be sufficient for a protective benefit in this model.

We hypothesized that COX-2 inhibition or deficiency mediates effects involved in the neuroprotection of the SNpc dopaminergic neurons in MPTP-induced mouse parkinsonism. This means that the differences in neuronal degeneration or microglial activation are not due to any effects of COX-2 inhibition on MPTP metabolism or MPP^+ ^accumulation but are due to inhibition of COX-2 mediated neurotoxicity. This is because it has been shown that the ratio between the amount of MPTP given and the amount of MPTP reaching the brain is constant and that concentrations of 1-methyl-4-phenylpyridinium (MPP^+^) in the striatum are similar regardless of age [40], with various kinds of COX-2 inhibitors used at different doses [1,27], or in COX-2 KO mice [1].

Based on earlier studies, we assume that microglial activation occurs before massive death of the dopaminergic neurons [41]. From our study, activated microglia appear in COX-2 KO and WT mice injected with saline, which demonstrates that COX-2 is not required for all microglial activation. We defined this as basal microglial activation; however, we did observe a significant reduction in MPTP-induced microglial activation in the COX-2 KO and inhibitor-treated mice relative to the MPTP-treated WT mice. COX-2 has been proposed to mediate microglial activation through the generation of reactive oxygen species [9]. Therefore, we speculate that COX-2 inhibition may mediate secondary microglia activation, which perpetuates the chronic inflammatory response seen in MPTP-induced PD. Studies from our group and from others have shown that activated microglia can cause dopaminergic neuronal death by releasing nitric oxide [10,14,42,43], superoxide free radicals [44], or proinflammatory cytokines like tumor necrosis factor-α [45]. Microglia can also induce neuritic beading [46] or synaptic stripping along dendrites [47] leading to synaptic disconnection and loss of trophic support and cell death [32,48]. Thus, activation of microglia may play an important role in secondary injury by releasing cytokines, reactive oxygen species, and nitric oxide which is important in the progressive loss of neurons and the perpetuation of the inflammatory response observed in PD. In microglial culture, COX-2 inhibitors reduce inducible nitric oxide synthase expression in lipopolysaccaride-activated microglia [49]; therefore reducing nitric oxide production, which suggests a positive modulatory effect of exogenous COX-2 inhibitor on activated microglial toxic substances release. The dopaminergic neurons of the SNpc are vulnerable to inflammation-induced oxidative stress because dopamine metabolism and autoxidation generate reactive oxygen species [50]. Consequently, the COX-2-mediated enzymatic reaction contributes to dopaminergic neuronal death by oxidizing dopamine to a reactive dopamine quinone [51], by increasing DNA oxidation [7], or through increased microglial activation leading to chronic inflammation.

To what extent is dopaminergic neuronal death attributable to microglial activation as opposed to a direct effect of cyclooxygenase-mediated reactions? Using the activated microglial inhibitor minocycline, Przedborski's group showed that activated microglia contribute to about 20% of the MPTP-induced TH-positive cell death [52]. The same group also showed 30–40% neuroprotection by the COX-2 inhibitor rofecoxib leaving 74–88% neuronal survival after MPTP injection but failed to show inactivation of microglia by COX-2 inhibition or deficiency [1]. The differences between their findings and ours may be due to different experimental settings, procedures, or technical variables, such as using different COX-2 inhibitors, pre-treatment time with the inhibitors, drug/toxin dosages, or unequal ages of mice. In addition, the previous work examined microglial activation at early stages following MPTP administration; thus, it is possible that examination of pathology at later stages is a better indicator of microglial activation. Moreover, a direct neurotoxic role of COX-2 activation cannot explain why COX-2 inhibitors may be protective or toxic in different PD models or systems [1,53]. As Wang et al. suggested, the final effect of inflammation may vary depending on the balance between neurotrophic and neurotoxic factors released by activated microglia in different systems or approaches, and the discrepancy thus may be due rather to an indirect role of COX-2 in neurotoxicity through regulation of inflammation [17]. The current result could be because of differences in the persistence of neuronal abnormalities or microglial activation.

**Figure 9:**
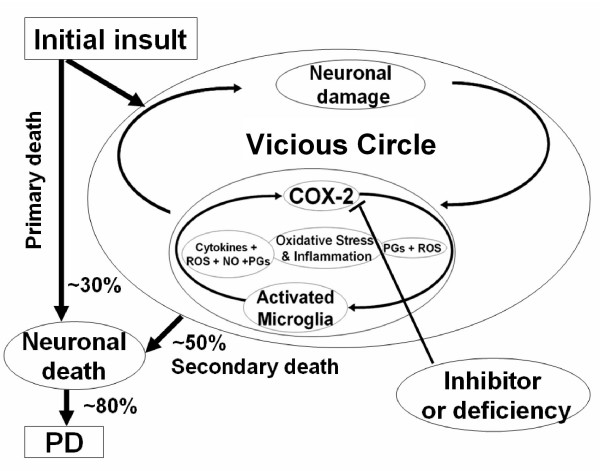
**Schematic flow chart depicting the role of the vicious circle in dopaminergic neurotoxicity. **For this study, the initial insult MPP^+ ^exerts direct dopaminergic neurodegeneration (~30% of the original numbers). Neuronal damage initiates the vicious circle with COX-2 and microglia as the key components feeding oxidative and inflammatory damage to the neurons, which in turn progresses to the secondary damage/death which is coupled to the release of factors that initiate another cycle of oxidative and inflammatory insults. The positive feed back loop may continue until the additional neuronal death (~50% of the remaining of the initial death) combined with the initial loss exceed the amount needed for normal motor control (~80% total loss); thus the PD symptoms, such as postural instability or hypokinesia, occurs. This vicious circle helps explain the chronic and prolong nature of PD progression. It is hypothesized that the blockade of COX-2 activity by selective COX-2 inhibitors or deficiency of COX-2 would attenuate the vicious circle and alleviate dopaminergic neurotoxicity by directly reducing COX-2 free radical production as well as by indirectly decreasing microglial activation and subsequent microglia-mediated damage.

Our study suggests a temporal and spatial relationship between microglial activation and neurodegeneration. Our data is supported by others who also reported this relationship which is accompanied by COX-2 induction [1,26,52]. This suggests that COX-2 may mediate microglial activation and play a key role in amplifying the inflammatory response and other toxic effects, which ultimately exacerbates dopaminergic neuronal loss. From Teismann et al. and Sugama et al[1,26], we can infer that COX-2 and activated microglia play an important role in secondary injury of dopaminergic neurons and in the perpetuation of inflammatory responses since their levels became noticeably high a few days after the MPP^+ ^exposure had already induced the primary loss of the dopaminergic neurons. Damaged neurons can activate microglia, and as Wu et al. showed, the blockade of microglial activation by minocycline prevents MPTP-induced neurotoxicity with evidence of reduced microglial-derived cytotoxic mediators, such as the formation of mature interleukin-1β, the activation of NADPH-oxidase and inducible nitric oxide synthase [52]. This suggests that microglial activation and release of toxic substances occurs before the secondary or progressive death of the dopaminergic neurons. Our data as well as those of Sugama et al. suggest that a prolonged oxidative and inflammatory environment, which follows the initial toxic insult, leads to the subsequent loss of neurons that have been compromised but may have potentially reversible damage [26]. In our MPTP paradigm, about 50% of the compromised dopaminergic neurons with reversible damage will die within one to two weeks of the initial injury. In this study, we believe that valdecoxib treatment rescues this population of compromised neurons, which is why we observed the neuroprotective properties afforded by valdecoxib (Fig. 9). Therefore, inhibition of COX-2 by valdecoxib or deficiency of COX-2 appears to be able both directly and indirectly to reduce dopaminergic neurodegeneration and progression to behavioral deficits induced by MPTP, possibly through the attenuation of microglia activation.

We have not repeated those experiments done by previous groups of investigators, which support our studies. From the previous works by Teismann et al. and others [1,26], we have not overlooked another possible interpretation of our results: that microglia may become inactive faster without COX-2. In general, activated immune cells including microglia become inactive over time through normal regulatory processes. Indeed, it has been shown that peak activation of microglia occurs around day 2–3 after MPTP injection, after which microglial activation dissipates [1,26]. Our results show extended activation of microglia two weeks after MPTP injection although this degree of activation is less than that seen during the first few days by other investigators. We also show much lesser amounts of activated microglia in COX-2-deficient or COX-2-inhibited mice. We conclude that COX-2 plays a role in sustaining microglial activation, or that activated microglia may be excluded persistent activation if COX-2 is lacking. In other words, our results showed that COX-2 inhibition or deficiency may be related to decreased microglia activation. With time, activation of microglia declines as COX-2 inhibition helps to reduce the perpetuation of a vicious circle that leads to chronic inflammation and secondary neurodegeneration.

## Conclusion

Our results provide strong support for the hypothesis that an exacerbated inflammatory process, potentially as a result of COX-2 mediated microglial activation, is detrimental to dopaminergic neurons; and that inhibition of COX-2 prevents progression of PD-like pathology and symptoms by breaking a vicious circle of perpetual microglial activation, thus producing the neuroprotective properties we observe. This is based on the strong correlations we find between COX-2 levels and microglial activation or dopaminergic neurodegeneration. We also present an alternative hypothesis that COX-2 inhibition or deficiency assists in attenuating microglial activation over time, which reduces the progression of the inflammatory response and reduces the perpetuation of the vicious circle instead of inhibiting microglial activation at the early stage when initial injury occurs. This alternative hypothesis does not affect our major point: that inhibiting COX-2 reduces the progression of the inflammatory response by breaking the vicious circle of dopaminergic neuronal cell death. This study suggests that COX-2 plays an important role in the secondary activation of microglia, in the progression of the inflammatory response, and in the progressive loss of the dopaminergic neurons in MPTP-induced PD. Therefore, COX-2 may serve as a potential target for the development of therapeutic strategies to treat the progressive cell loss observed in PD.

## Competing interests

The author(s) declare that they have no competing interests.

## Authors' contributions

RV participated in the project design, animal management, all experimental procedures, statistical analysis and manuscript preparation. ML participated in histological procedures. DYC participated in MPTP administration procedure. XN helped to draft the manuscript. RLH helped to draft the manuscript. GB conceived of the study and participated in its design and coordination and helped to draft the manuscript. All authors read and approved the final manuscript.
